# Exome sequencing in fetuses with short long bones detected by ultrasonography: A retrospective cohort study

**DOI:** 10.3389/fgene.2023.1032346

**Published:** 2023-02-27

**Authors:** Yanlin Huang, Chang Liu, Hongke Ding, Yunan Wang, Lihua Yu, Fangfang Guo, Fake Li, Xiaomei Shi, Yan Zhang, Aihua Yin

**Affiliations:** Medical Genetic Center, Guangdong Women and Children Hospital, Guangzhou, Guangdong, China

**Keywords:** fetal short long bones, skeletal dysplasia, exome sequencing, ultrasound examination, prenatal diagnosis

## Abstract

**Background:** Prenatal diagnosis of fetal short long bones (SLBs) was reported to be associated with skeletal dysplasias, chromosomal abnormalities, and genetic syndromes. This study aims to identify the genetic causes for fetal short long bones, and retrospectively evaluate the additional diagnostic yield of exome sequencing (ES) for short long bones following the use of conventional genetic testing.

**Methods:** A cohort of ninety-four fetuses with sonographically identified short long bones was analyzed by trio-exome sequencing between January 2016 and June 2021. Fetuses with abnormal results of karyotype or chromosomal microarray analysis were excluded. Variants were interpreted based on ACMG/AMP guidelines. All diagnostic *de novo* variants were validated by Sanger sequencing.

**Results:** Of the 94 fetuses, 38 (40.4%) were found to carry causal genetic variants (pathogenic or likely pathogenic) in sixteen genes with 38 variants. Five fetuses (5.3%) had variant(s) of uncertain significance. Thirty-five cases (37.2%) were diagnosed as genetic skeletal dysplasias including 14 different diseases that were classified into 10 groups according to the Nosology and Classification of Genetic Skeletal Disorders. The most common disease in the cohort was achondroplasia (28.9%), followed by osteogenesis imperfecta (18.4%), thanatophoric dysplasia (10.5%), chondrogenesis (7.9%), and 3-M syndrome (5.3%). The diagnostic yield in fetuses with isolated short long bones was lower than the fetuses with non-isolated short long bones, but not reached statistical significance (27.3% vs. 44.4%; *p* = 0.151). Whereas, the rate in the fetuses with other skeletal abnormalities was significantly higher than those with non-skeletal abnormalities (59.4% vs. 32.5%, *p* = 0.023), and the diagnostic rate was significantly higher in femur length (FL) below -4SDs group compared with FL 2-4SDs below GA group (72.5% vs. 16.7%; *p* < 0.001). A long-term follow-up showed that outcomes for fetuses with FL 2-4SDs below GA were significantly better than those with FL below -4SDs. Additionally, fourteen (36.8%) novel short long bones-related variants were identified in the present study.

**Conclusion:** The findings suggest that in fetuses with short long bones routine genetic tests failed to determine the underlying causes, exome sequencing could add clinically relevant information that could assist the clinical management of pregnancies. Novel pathogenic variants identified may broaden the mutation spectrum for the disorders and contributes to clinical consultation and subsequent pregnancy examination.

## 1 Introduction

Routine fetal biometric evaluation includes femur diaphysis length measurement (Salomon et al., 2019). In cases where the femur length (FL) is 2 or more standard deviations (SDs) below the normal range, the guidelines recommend measuring the length of the other long bones and performing a thorough skeletal assessment (Beke et al., 2005; Speer et al., 2014; Rao and Platt, 2016; Tzadikevitch et al., 2021). Short long bones may be constitutional and/or attributed to race, ethnicity and familial tendency (Liu et al., 2019; Tzadikevitch et al., 2021). When no coexistence of other fetal abnormalities or abnormal uterine artery Doppler flows is demonstrated, a good pregnancy outcome may be expected (Zalel et al., 2002; Mohr-Sasson et al., 2020). Whereas, prenatal diagnosis of fetal SLBs was reported to be associated with skeletal dysplasias, chromosomal abnormalities and genetic disorders (Zalel et al., 2002; Papageorghiou et al., 2008; Kaijomaa et al., 2016; Mohr-Sasson et al., 2020). In some cases, it may be a sign of intrauterine growth restriction (FGR) and placental insufficiency, resulting in an increased risk for pregnancy complications, including pre-eclampsia, placental abruption and intrauterine fetal death (Weisz et al., 2008; Aviram et al., 2015; Kaijomaa et al., 2016; Tzadikevitch et al., 2021). According to the latest version of the Nosology, skeletal dysplasia comprises 461 different diseases that are classified into 42 groups based on their clinical, radiographic, and molecular phenotypes. Remarkably, 437 skeletal dysplasia-related genes are functionally diverse, involved in a broad range of cell biologic processes, and cause diseases by a variety of mutational mechanisms (Mortier et al., 2019). Pathogenic variants in *FGFR3*, *COL1A1*, *COL1A2*, and *COL2A1* are common causes of isolated short stature or osteogenesis imperfecta. However, features of skeletal dysplasias such as epiphyseal stippling, bowing of the long bones, aberrant bone mineralization, and fractures are not always identified on antenatal scanning (Ramachandrappa et al., 2018; Victoria et al., 2018). Furthermore, disproportionate limb development may be a late sign as exemplified by achondroplasia where SLBs are rarely detected before the third trimester (Kurtz et al., 1986; Patel et al., 1995; Ramachandrappa et al., 2018). The complex etiology, combined with the highly variable and often overlapping presentations of skeletal dysplasias and SLBs, challenge the ability of traditional prenatal diagnosis.

The conventional molecular diagnosis strategies mainly involved karyotyping or chromosomal microarray analysis (CMA). Karyotyping has a diagnostic yield of about 13%–16% in fetuses with sonographically identified SLBs, and CMA provides an additional diagnostic yield of 6%–27% (Gaudry et al., 2012; Liu et al., 2019; Hu et al., 2021; Tzadikevitch et al., 2021). In the last decade, next-generation sequencing (NGS) has revolutionized the genetic testing of diseases with high genetic and allelic heterogeneity, such as skeletal dysplasias, allowing hundreds of genes to be screened simultaneously. Based on the statements released by the International Society for Prenatal Diagnosis (ISPD), the Society for Maternal Fetal Medicine (SMFM), the Perinatal Quality Foundation (PQF), and the American College of Medical Genetics and Genomics (ACMG), NGS can be used with ultrasound anomalies when standard diagnostic genetic testing, such as CMA, failed to yield a definitive diagnosis (Lord et al., 2019; Tang et al., 2021). In addition, SLBs are mainly attributed to monogenic disorders, such as skeletal dysplasias (Mortier et al., 2019), and therefore NGS may be of importance in their prenatal evaluation. Especially, if a specific diagnosis is suspected or when FL is > 4 SDs below the mean, targeted capture sequencing or whole exome sequencing should be performed (Liu et al., 2019; Tang et al., 2021). Definitive molecular diagnosis can provide information about prognosis of the disease and treatment regimens, as well as the estimation of the likelihood of recurrence.

There is limited data in the literature regarding ES findings in fetuses with SLBs to evaluate its clinical value. In the present study, ES was performed to identify genetic causes for 94 fetuses with sonographically identified SLBs, and to evaluate the diagnostic yield of ES for fetal SLBs. Besides of genetic diagnosis, we also performed long-term follow-ups to assess the prognoses and outcomes in fetuses with SLBs.

## 2 Materials and methods

### 2.1 Ethics statement

This retrospective cohort study was conducted at Guangdong Women and Children Hospital. The study has been approved by our institutional review board and clinical research ethics committee. Written informed consents were obtained from all participants. Authors had access to information that could identify individual participants, and the information was anonymized prior to submission. All the procedures performed in the study were in accordance with the Declaration of Helsinki and as we previously described (Liu et al., 2022).

### 2.2 Subject identification and enrollment

**FIGURE 1:**
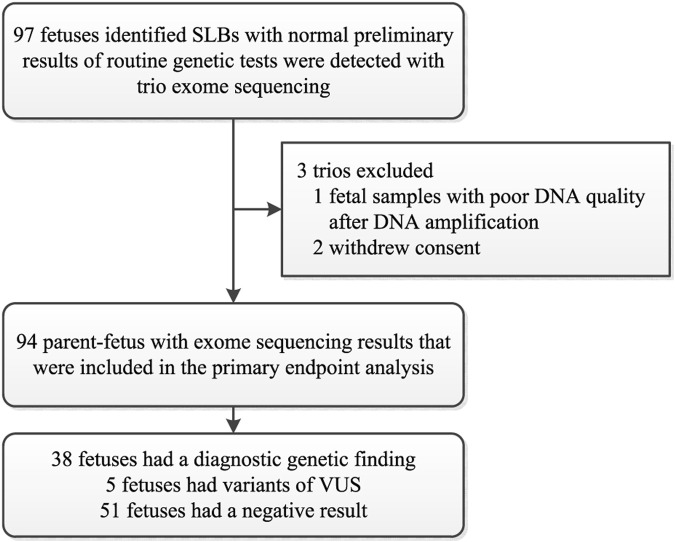
The flow diagram of the cohort and outcomes.

Between January 2016 and June 2021, a cohort of 97 couples consented to further ES with normal preliminary results of routine genetic tests. Apart from 2 couples who withdrew consent and one sample that was not of good enough quality for analysis, DNA samples from 94 eligible trios were therefore used for the analysis of the primary outcome (Figure 1). The sonographic criteria include the presence of short limb deformities in which fetal femur length and/or other long bones < −2 SD of our reference ranges at mid-trimester ultrasonography with/without other abnormalities (Papageorghiou et al., 2008). The traditional molecular diagnosis strategies include karyotyping and CMA. Fetuses with chromosomal aneuploidies or CNV abnormalities were excluded. The isolated-SLBs group includes fetuses with short long bones only, while the non-isolated-SLBs group includes fetuses with SLBs and another abnormality. To compare the degree of femur shortening, Z-score was calculated as (X_GA_ − M_GA_)/SD_GA_, where X_GA_ is the ultrasound measurement of the fetal femur, and M_GA_ and SD_GA_ are the mean value and standard deviation at the corresponding gestational age (GA) in weeks (Sananes et al., 2009). The clinical details of enrolled fetuses are summarized in Table 1 and Additional file 1 (Supplementary Table S1). The chorionic villi, amniotic fluid, umbilical cord blood or skin tissue of aborted fetuses, and peripheral blood of family members were collected by maternal-fetal medicine specialists at our center through the workup already being performed as part of the standard-of-care. Pretest counseling for prenatal ES was delivered in an intelligible fashion to every enrolled family by trained genetic professionals. Results were reported to the parents after a multidisciplinary team of clinical and laboratory geneticists, obstetricians, and genetic counselors reviewed all the variants related to the ultrasound anomalies.

**TABLE 1:** Prenatal phenotype and genotype information of the genetically diagnosed fetuses in the cohort.

Case	Sample	MA(y)	GA(w)	Family history	Ultrasound findings		Clinical diagnosis	Exome sequencing results			Pregnancy outcome	Follow-up
					Skeletal anomalies	Other anomalies		ACMG Level	HGVS	Heterozygosity/Inheritance		
(A) Patients Diagnosed with Pathogenic or Likely Pathogenic Variants												
1	ST	24	29+	No	SLBs with fracture, angulated humerus	No	OI1/3	PVS1+PS2+PM2_sup = P	*COL1A1*(NM_000088):c.957 + 1G>T	Het/AD/*de novo*	TOP	-
2	UCB	31	25+	No	SLBs, bowing of the legs	No	OI1/3	PS2+PS4+PM2_sup + PP2+PP3 = P	*COL1A1*(NM_000088):c.3118G>A(p.G1040S)	Het/AD/*de novo*	TOP	-
3	UCB	24	28+	No	SLBs, femur fracture, tibial bowing	No	OI1/3	PS2+PS4_sup + PM2_sup + PP2+PP3 = LP	*COL1A2*(NM_000089):c.3277G>A(p.G1093S)	Het/AD/Pat	TOP	-
4	AF	25	32	No	SLBs	No	OI1/3	PS4+PS3+PS2+PM2_sup = P	*COL1A2*(NM_000089):c.1801G>A(p.G601S)	Het/AD/*de novo*	TOP	-
5	UCB	23	31+	No	SLBs, femoral bowing	No	OI1/3	PS4+PS3+PS2+PM2_sup = P	*COL1A2*(NM_000089):c.3034G>A(p.G1012S)	Het/AD/*de novo*	TOP	Pathological confirmed
6	UCB	35	25+	No	SLBs, talipes equinovarus	Micrognathia, abnormal posturing of the arms	ACG2	PS2+PM2_sup + PM5+PP2+PP3 = LP	*COL2A1*(NM_001844):c.1439G>A(p.G480E)	Het/AD/*de novo*	TOP	-
7	UCB	28	30	No	SLBs	No	ACG2	PS2+PM2_sup + PP2+PP3 = LP	*COL2A1*(NM_001844):c.3580G>C(p.G1194R)	Het/AD/*de novo*	TOP	-
8	AF	28	24	No	SLBs	Cleft palate	ACG2	PS2+PM2_sup + PM5+PP2+PP3 = LP	*COL2A1*(NM_001844):c.3472G>A(p.G1158S)	Het/AD/*de novo*	TOP	-
9	UCB	34	29	No	SLBs	No	3M1	PVS1+PM2_sup = LP; PVS1+PS3+PM2_sup = P	*CUL7*(NM_001168370):c.4855C>T(p.Q1619*); c.4429NoC>T(p.R1477*)	Het/AR/Pat; Het/AR/Mat	TOP	-
10	UCB	26	25	Previous pregnancy with SLBs, narrow thorax and pulmonary hypoplasia	SLBs	Increased nuchal translucency (4.2 mm)	3M1	PVS1+PM2_sup = LP; PVS1+PM2_sup = LP	*CUL7*(NM_001168370):c.2260C>T(p.R754*); c.3333del(p.F1112Sfs*61)	Het/AR/Pat; Het/AR/Mat	TOP	Pathological confirmed
11	ST	34	14+	No	SLBs, absence of the fibula	Tricuspid regurgitation	SRTD3	PVS1+PM2_sup = LP; PM3_str + PM2_sup + PP3 = LP	*DYNC2H1*(NM_001080463):c.6808C>T(p.R2270*); c.4267C>T(p.R1423C)	Het/AR/Pat; Het/AR/Mat	TOP	-
12	ST	30	30+	No	Hypoplasia of the nasal bone, narrow thorax, SLBs, spinal dysplasia	Thickened skin	CDPX2	PS2+PM2_sup + PP2+PP3 = LP	*EBP*(NM_006579):c.214T>C(p.C72R)	Hem/XLD/*de novo*	TOP	Pathological confirmed
13	UCB	22	U	No	SLBs, femoral bowing	Enlarged kidneys, trigonocephaly	ACH	PS1+PS2+PS3+PS4+PM2_sup = P	*FGFR3*(NM_000142):c.1138G>A(p.G380R)	Het/AD/*de novo*	TOP	-
14	AF	30	18	No	SLBs	Increased nuchal translucency (3.2 mm)	ACH	PS1+PS2+PS3+PS4+PM2_sup = P	*FGFR3*(NM_000142):c.1138G>A(p.G380R)	Het/AD/*de novo*	TOP	-
15	UCB	21	23	No	SLBs, narrow thorax	Increased nuchal translucency (3.3 mm), cerebellar dysplasia	TD1	PS2+PS3+PS4+PM2_sup = P	*FGFR3*(NM_000142):c.746C>G(p.S249C)	Het/AD/*de novo*	TOP	-
16	AF	29	26	No	FGR, SLBs	No	ACH	PS1+PS2+PS3+PS4+PM2_sup = P	*FGFR3*(NM_000142):c.1138G>A(p.G380R)	Het/AD/*de novo*	TOP	-
17	AF	25	33	No	FGR, SLBs	Polyhydramnios	ACH	PS1+PS2+PS3+PS4+PM2_sup = P	*FGFR3*(NM_000142):c.1138G>A(p.G380R)	Het/AD/*de novo*	TOP	-
18	UCB	30	27+	No	SLBs	No	ACH	PS1+PS2+PS3+PS4+PM2_sup = P	*FGFR3*(NM_000142):c.1138G>A(p.G380R)	Het/AD/*de novo*	TOP	Pathological confirmed
19	UCB	33	31	No	Short femur	No	HCH	PS1+PS2+PS3+PS4+PM2_sup + PM5 = P	*FGFR3*(NM_000142):c.1620C>A(p.N540K)	Het/AD/*de novo*	TOP	-
20	UCB	33	30	No	SLBs	No	ACH	PS1+PS2+PS3+PS4+PM2_sup = P	*FGFR3*(NM_000142):c.1138G>A(p.G380R)	Het/AD/*de novo*	TOP	-
21	UCB	30	37	No	SLBs	No	ACH	PS1+PS2+PS3+PS4+PM2_sup = P	*FGFR3*(NM_000142):c.1138G>A(p.G380R)	Het/AD/*de novo*	TOP	-
22	UCB	25	30+	No	SLBs, cerebellar hypoplasia	No	ACH	PS1+PS2+PS3+PS4+PM2_sup = P	*FGFR3*(NM_000142):c.1138G>A(p.G380R)	Het/AD/*de novo*	TOP	-
23	AF	25	20+	No	SLBs	No	TD1	PS2+PS3+PS4+PM2_sup = P	*FGFR3*(NM_000142):c.742C>T(p.R248C)	Het/AD/*de novo*	TOP	-
24	UCB	37	29+	No	SLBs, femoral bowing	No	ACH	PS1+PS2+PS3+PS4+PM2_sup = P	*FGFR3*(NM_000142):c.1138G>A(p.G380R)	Het/AD/*de novo*	TOP	-
25	ST	24	36+	No	SLBs, femoral bowing	No	ACH	PS1+PS2+PS3+PS4+PM2_sup = P	*FGFR3*(NM_000142):c.1138G>A(p.G380R)	Het/AD/*de novo*	TOP	Pathological confirmed
26	AF	32	27	No	SLBs, femoral bowing	No	TD1	PS2+PS3+PS4+PM2_sup = P	*FGFR3*(NM_000142):c.1118A>G(p.Y373C)	Het/AD/*de novo*	TOP	-
27	UCB	24	23	No	SLBs, dumbbell-shaped femur	No	TD1	PS2+PS3+PS4+PM2_sup = P	*FGFR3*(NM_000142):c.742C>T(p.R248C)	Het/AD/*de novo*	TOP	-
28	UCB	26	31	No	SLBs	Polyhydramnios	ACH	PS1+PS2+PS3+PS4+PM2_sup = P	*FGFR3*(NM_000142):c.1138G>A(p.G380R)	Het/AD/*de novo*	TOP	-
29	UCB	38	27+	One previous pregnancy with the same anomalies	Bilateral talipes equinovarus, short and bowed humerus, relatively short spine	Cleft palate	LRS	PS4+PM2_sup + PP3 = LP	*FLNB*(NM_001164317):c.4664G>A(p.G1555D)	Het/AD/Mat	TOP	-
30	UCB	28	21	No	Short femur, femoral bowing, angulated ulna and radius	Intracranial cystic lesion, oligohydramnios	PHS	PVS1+PS2+PM2_sup = P	*GLI3*(NM_000168):c.3172C>T(p.R1058*)	Het/AD/*de novo*	TOP	-
31	UCB	34	30	No	FGR, SLBs	Umbilical vein varix	BBSOAS	PVS1+PS2+PM2_sup = P	*NR2F1*(NM_005654):c.1117C>T(p.R373*)	Het/AD/*de novo*	TOP	-
32	ST	28	19	Previous pregnancy with skull deformation and SLBs	SLBs, dumbbell-shaped femur	No	OI9	PM2_sup + PM3+PS3_sup + PP3+PP4 = LP	*PPIB*(NM_000942):c.509G>A(p.G170D)	Hom/AR/Pat/Mat	TOP	Pathological confirmed
33	ST	30	20	One previous pregnancy with the same anomalies	SLBs, femoral bowing, angulated tibia and fibula		OI9	PM2_sup + PM3+PS3_sup + PP3+PP4 = LP	*PPIB*(NM_000942):c.509G>A(p.G170D)	Hom/AR/Pat/Mat	TOP	Pathological confirmed
34	ST	27	21+	No	SLBs, spina bifida occulta	Agenesis of corpus callosum; Cerebellar dysplasia; Cleft palate; Echogenic fetal bowel	ARCL2B	PM2_sup + PM3_str + PP3+PP4 = LP; PVS1+PM2_sup = LP	*PYCR1*(NM_006907):c.575G>T(p.G192V); c.345del(p.R116Gfs*6)	Het/AR/Pat; Het/AR/Mat	TOP	-
35	UCB	22	26+	Previous pregnancy with tetralogy of Fallot	SLBs	Thickened nuchal skin fold, ventricular hypertrophy, battledore placenta	NS5	PS2+PS3+PS4+PM2_sup + PM5 = P	*RAF1*(NM_002880):c.770C>T(p.S257L)	Het/AD/*de novo*	Died After Birth	Died at 2 months
36	UCB	34	29+	No	SLBs	No	MCOPS12	PS2+PM2_sup + PM5+PP3 = LP	*RARB*(NM_000965):c.1160G>A(p.R387H)	Het/AD/*de novo*	TOP	-
37	UCB	27	33	No	SLBs	Dilated fourth ventricle; Cleft palate; Atrial septal defect and ventricular septal defect; Duplication of renal pelvis; Echogenic fetal bowel; Single umbilical artery	CSS4	PS2+PS4+PM2_sup + PM5+PP3 = P	*SMARCA4*(NM_001128849):c.2653C>T(p.R885C)	Het/AD/*de novo*	TOP	-
38	UCB	25	28	The pregnant woman with spondyloepiphyseal dysplasia and scoliosis	Abnormality of the vertebral column, short and curved long bones	No	SMDK	PS2+PS4+PM2_sup + PM4 = P	*TRPV4*(NM_021625):c.1412_1414del(p.F471del)	Het/AD/Mat	TOP	-
**(B) Patients Suspiciously Diagnosed with Variants of Uncertain Significance**												
39	UCB	29	25+	Previous pregnancy with cardiomegaly and celiac effusion	SLBs	Cardiomegaly; Pericardial effusion; Celiac effusion; Echogenic fetal bowel; Polyhydramnios	CDAN2	PM2_sup + PM4+PP4 = VUS; PM2_sup + PP3+PP4 = VUS	*SEC23B*(NM_001172745):c.1327_1338del(p.G443_S446del); c.466A>G(p.S156G)	Het/AR/Pat; Het/AR/Mat	TOP	-
40	AF	27	17+	No	SLBs, limited knee flexion, bilateral talipes equinovarus, anomaly of the sacral spine	Increased nuchal translucency (3.6 mm); Polyhydramnios	DBQD2	PM2_sup + PP3+PP4 = VUS	*XYLT1*(NM_022166):c.2456G>T(p.G819V)	Hom/AR/Pat/Mat	TOP	-
41	UCB	30	33	No	SLBs	Tricuspid regurgitation	WRWFFR	PM2_sup + PP3+PP4 = VUS	*ZC4H2*(NM_018684):c.635C>T(p.S212F)	Hem/XLD/*de novo*	Alive	Healthy
42	UCB	38	28	No	SLBs, narrow thorax	Ventricular septal defect	SRTD3	PM2_sup + PP3+PP4 = VUS; PM2_sup + PP3+PP4 = VUS	*DYNC2H1*(NM_001080463):c.9977G>C(p.R3326P); c.10526T>G(p.M3509R)	Het/AR/Pat; Het/AR/Mat	Unknown	Refuse follow-up
43	AF	29	31+	No	SLBs	No	SJS1	PM2_sup + PM4+PP4 = VUS; PM2_sup + BP4+PP4 = VUS	*HSPG2*(NM_005529):c.2397_2399del(p.F800del); c.2008G>A(p.V670I)	Het/AR/Pat; Het/AR/Mat	TOP	-

SLBs, short long bones; MA, maternal age; GA, gestational age; UCB, umbilical cord blood; AF, amniotic fluid; ST, skin tissue; TOP, termination of pregnancy; P, pathogenic; LP, likely pathogenic; VUS, variants of uncertain significance; AD, autosomal dominant; AR, autosomal recessive; XLD, X-linked dominant; Hom, homozygosity; Het, heterozygosity; Hem, hemizygosity; Pat, paternal; Mat, maternal; sup, supporting; str, strong. ACH, achondroplasia; OI1/3, osteogenesis imperfecta types I or III; TD1, thanatophoric dysplasia, type I; ACG2, achondrogenesis, type II; 3M1, 3-M syndrome 1; OI9, osteogenesis imperfecta, type IX; BBSOAS, Bosch-Boonstra-Schaaf optic atrophy syndrome; CDPX2, chondrodysplasia punctata, X-linked dominant; CSS4, Coffin-Siris syndrome 4; ARCL2B, cutis laxa, autosomal recessive, type IIB; HCH, hypochondroplasia; LRS, Larsen syndrome; MCOPS12, microphthalmia, syndromic 12; NS5, Noonan syndrome 5; PHS, Pallister-Hall syndrome; SRTD3, short-rib thoracic dysplasia 3 with or without polydactyly; SMDK, spondylometaphyseal dysplasia, Kozlowski type; CDAN2, Dyserythropoietic anemia, congenital, type II; DBQD2, Desbuquois dysplasia 2; WRWFFR, Wieacker-Wolff syndrome, female-restricted; SJS1, Schwartz-Jampel Syndrome Type 1.

### 2.3 Exome sequencing

**FIGURE 2:**
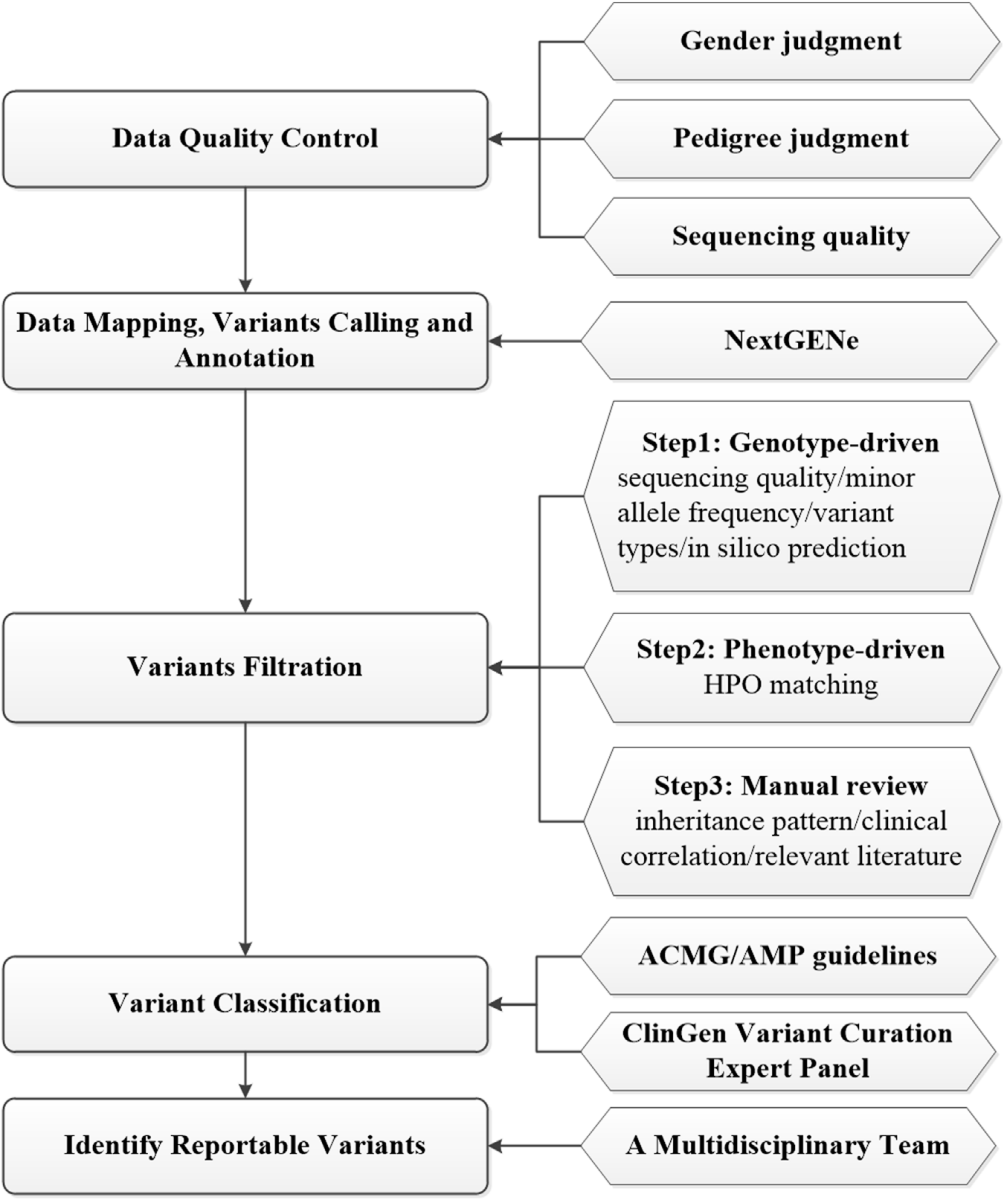
Data analysis process of exome sequencing.

Ninety-four fetuses received genetics evaluations by using prenatal ES, as previously described (Zheng et al., 2018; Dai et al., 2019; Han et al., 2020; Zhang B et al., 2020; Liu et al., 2022). Genomic DNA was extracted by using the SolPure Blood DNA kit (Magen, Guangzhou, China) according to the manufacturer’s instructions and was fragmented by Q800R Sonicator (Qsonica, CT, United States). The paired-end libraries were prepared following the Illumina library preparation protocol. Custom-designed NimbleGen SeqCap probes (Roche NimbleGen, Madison, WI) were used for in-solution hybridization to enrich target whole-exome sequences for WES or target sequences included ∼5,000 genes potentially associated with known Mendelian genetic diseases for clinical exome sequencing (CES) (AmCare Genomic Laboratory, Guangzhou, China). Captured DNA samples were amplified by PCR with indexed primers and then sequenced on MGISEQ-2000RS, DNBSEQ-T7 sequencers (MGI, ShenZhen, China) or NovaSeq 6000 sequencers (Illumina, San Diego, CA) with pair-end 150 base pairs (bp) model following the manufacturer’s protocol. Low-quality reads (Phred score < Q20) were removed before demultiplexing. An overview of prenatal ES data analysis and interpretation process is summarized in Figure 2. Before bioinformatics analysis, data quality control would be conducted based on gender judgment, pedigree judgment, and data volume judgment. Raw fastq reads were filtered by using Fastp (v0.23) to remove low quality and adapter contaminated reads, leaving clean paired-end reads were aligned to the GRCh37/hg19 human reference sequence by NextGENe software (SoftGenetics, State College, PA). Then, NextGENe software was used for identifying single-nucleotide variants (SNVs) and small insertions/deletions (InDels) by using the recommended standard settings. Finally, variants were annotated by NextGENe software with multiple public database and in-house database (Figure 2).

### 2.4 Variants filtration and evaluation

Quality control for each sample included an average depth of >100X and >95% targeted region with at least 20X in the cohort. The annotated variants were filtrated in a stepwise model as shown in Figure 2. The first genotype-driven step prioritized the variants based on sequencing quality, minor allele frequency, variant types and *in silico* prediction without phenotypic data. All the annotated variants, excluding low quality ones (with a depth <10X or alternate allele proportion <0.20) were subject to downstream analysis. Variants with a minor allele frequency (MAF) > 0.5% in the Genome Aggregation Database (GnomAD, https://gnomad.broadinstitute.org/) were filtered out except for those in ClinVar (https://www.ncbi.nlm.nih.gov/clinvar/), HGMD (http://www.hgmd.cf.ac.uk/) and ClinGen BA1 exception list (Ghosh R et al., 2018). Next, we mainly focused on genomic regions known or likely associated with the disease and potential protein-altering variants (e.g., missense, start loss, stop gain/loss, frameshift, in-frame insertion/deletion, or canonical splice-site) were retained. Variants were evaluated with the predictors included in the Varsome website (https://Varsome.com/), VarSEAK website (https://varseak.bio/), and SpliceAI software (Jaganathan et al., 2019). In the second phenotype-driven analysis step, the rare variants that potentially related to initial clinical indications for prenatal diagnosis was evaluated with the aid of HPO matching. In the third analysis step, the remaining variants were closely reviewed by trained genetic professionals based on inheritance pattern, clinical correlation and relevant literature. Search engines such as Mastermind (https://mastermind.genomenon.com/) and PubMed (https://pubmed.ncbi.nlm.nih.gov/) were used for retrieving published literature on variant-related case reports. The variant number in each trio after each screening step was shown in Additional file 2 (Supplementary Table S2). The information for all variants passing each screening step was shown in Additional file 3 (Supplementary Table S3). Candidate variants were classified according to the American College of Medical Genetics and Genomics/Association for Molecular Pathology (ACMG/AMP) guidelines (Richards et al., 2015) and ClinGen variant curation expert panel guidelines (Abou et al., 2018; Brnich et al., 2019). A multidisciplinary team of clinical and laboratory geneticists, obstetricians and genetic counselors reviewed all the rare phenotype-related variants to identify the reportable variants. All diagnostic *de novo* variants were validated by Sanger sequencing. The primer sequences of de novo variants were shown in Additional file 4 (Supplementary Table S4).

### 2.5 Statistical analysis

Continuous data (maternal age, GA at initial diagnosis) are presented as mean (standard deviation) and categorical variables are presented as number (percentage). Comparison of diagnostic yield of ES between groups was conducted using the chi-square test or Fisher’s exact test. A two-sided *p* < 0.05 was considered statistically significant. All statistical analyses were performed using SPSS Statistics 21.0 (SPSS, Chicago, IL, United States).

## 3 Results

### 3.1 Clinical descriptions

A total of 94 parent-fetuses were enrolled in the current study. All of the enrolled fetuses underwent detailed ultrasound examinations during pregnancy, and the clinical manifestations were summarized in Supplementary Table S1 and Table 1. Maternal age ranged from 21 to 44 years (29.46 ± 4.48 years). GA at initial diagnosis ranged from 14^+5^ to 36^+6^ weeks (27.51 ± 4.38 weeks). Thirteen cases had a history of adverse pregnancy outcomes, and 9 of those were associated with skeletal dysplasias. Twenty-two fetuses had an ultrasonic diagnosis with isolated SLBs, 32 with SLBs combined with other skeletal abnormalities, and 40 with SLBs combined with non-skeletal abnormalities. Moreover, FL in 42.6% (40/94) of the fetuses were below -4SDs (Table 2).

**TABLE 2:** The yield of exome sequencing according to clinical parameters.

Group	Total cases	Cases with P/LP	% Of P/LP	Cases with VUS	% Of VUS	Cases with abnormal results	% Of abnormal results	*p*-value^a^	*P* ^b^ value^c^
Total	94	38	40.43	5	5.32	43	45.74		
SLBs Category									
Isolated SLBs	22	6	27.27	1	4.55	7	31.82	0.151	0.134
Non-isolated SLBs	72	32	44.44	4	5.56	36	50.00		
with other skeletal anomalies	32	19	59.38	2	4.55	21	65.63	0.023	0.018
with non-skeletal anomalies	40	13	32.50	2	5.00	15	37.50		
Z-score									
−2 > Z-score ≥ −4	54	9	16.67	5	9.26	14	25.93	<0.001	<0.001
Z-score < −4	40	29	72.50	0	0.00	29	72.50		
Family history									
Yes	13	6	46.15	1	7.69	7	53.85	0.650	0.528
No	81	32	39.51	4	4.94	36	44.44		
Maternal age									
<35 years	83	35	42.17	4	4.82	39	46.99	0.536^b^	0.506
≥35 years	11	3	27.27	1	9.09	4	36.36		
GA at initial diagnosis									
<27^+6^ GW (Second trimester)	48	18	37.50	2	4.17	20	41.67	0.555	0.418
≥28^+0^ GW (Third trimester)	46	20	43.48	3	6.52	23	50.00		
Sample Type									
Amniotic fluid	30	7	23.33	2	6.67	9	30.00	—	—
Umbilical cord blood	53	24	45.28	3	5.66	27	50.94		
Chorionic villus	1	0	0.00	0	0.00	0	0.00		
Skin tissue	10	7	70.00	0	0.00	7	70.00		
Pregnancy outcome^b^									
Termination of pregnancy	63	37	58.73	3	4.76	40	63.49	<0.001	<0.001
Dead in uterus	1	0	0.00	0	0.00	0	0.00		
Dead after birth	1	1	100.00	0	0.00	1	100.00		
Alive	28	0	0.00	1	3.57	1	3.57		

P, pathogenic; LP, likely pathogenic; VUS, variants of uncertain significance; SLBs, short long bones; GA, gestational age; GW, gestational week.

^a^ Comparison of diagnostic rate of “Cases with P/LP” between groups.

^b^ 1 case refused follow-up, *p*-value for “Termination of pregnancy” vs. “Alive” comparison.

^c^ Comparison of diagnostic rate of “Cases with abnormal results” between groups.

### 3.2 Diagnosis yields of Trio-ES

Of the 94 fetuses, 38 (40.4%) were found to carry causal genetic variants (pathogenic or likely pathogenic) in sixteen genes. Five (5.3%) fetuses had variant(s) of uncertain significance (VUS), thereby yielding a total detection rate of 45.7%. The diagnostic rate in fetuses with isolated SLBs was lower than those with non-isolated SLBs, but not reached statistical significance (27.3% vs. 44.4%; *p* = 0.151). While the yield of ES among the fetuses with other skeletal abnormalities was significantly higher than those with non-skeletal abnormalities (59.4% vs. 32.5%; *p* = 0.023). Moreover, the diagnostic rate was 58.7% among terminated fetuses, which was much higher than those in alive fetuses (3.6%, *p* < 0.001). Fetuses with Z-scores below −4SDs show a significantly higher diagnostic yield than the fetuses with Z-scores between −2SDs and −4SDs (72.5% vs. 16.7%; *p* < 0.001). However, we did not find any correlation between the yield of ES and family history, maternal age, sample type, or gestational week at initial diagnosis (Table 2).

### 3.3 Etiologic diagnosis

The etiologic diagnosis has been identified for 38 cases. These were linked to *FGFR3* (16 cases), *COL1A2* (3 cases), *COL2A1* (3 cases), *COL1A1* (2 cases), *CUL7* (2 cases), and one case in each of the *PPIB*, *EBP, FLNB, GLI3, NR2F1, PYCR1, RAF1, RARB, DYNC2H1, SMARCA4,* and *TRPV4* genes. 35 cases (37.2%) were diagnosed as genetic skeletal dysplasias including 14 different diseases that are classified into 10 groups according to the 2019 revision of the Nosology and Classification of Genetic Skeletal Disorders (Table 3). Three other cases were diagnosed with Noonan syndrome 5 (NS5), Microphthalmia, syndromic 12 (MCOPS12), and Bosch-Boonstra-Schaaf optic atrophy syndrome (BBSOAS) respectively. Among genetic skeletal dysplasias in our cohort, the most common disease is achondroplasia (28.9%), followed by osteogenesis imperfecta (18.4%), thanatophoric dysplasia (10.5%), chondrogenesis (7.9%), and 3-M syndrome (5.3%). One case in each of other skeletal dysplasias are shown in Table 3.

**TABLE 3:** Etiologic diagnosis and categories for 38 fetuses with genetic disorders.

ID	Disease	Abbreviation	Gene	N (%)	Group^a^
1	Achondroplasia	ACH	*FGFR3*	11(28.9)	*FGFR3* chondrodysplasia group
2	Thanatophoric dysplasia, type I	TD1	*FGFR3*	4(10.5)	*FGFR3* chondrodysplasia group
3	Hypochondroplasia	HCH	*FGFR3*	1(2.6)	*FGFR3* chondrodysplasia group
4	Osteogenesis imperfecta types I or III	OI1/3	*COL1A1/2*	5(13.2)	Osteogenesis Imperfecta and decreased bone density group
5	Osteogenesis imperfecta, type IX	OI9	*PPIB*	2(5.3)	Osteogenesis Imperfecta and decreased bone density group
6	Cutis laxa, autosomal recessive, type IIB	ARCL2B	*PYCR1*	1(2.6)	Osteogenesis Imperfecta and decreased bone density group
7	Achondrogenesis, type II	ACG2	*COL2A1*	3(7.9)	Type 2 collagen group
8	3-M syndrome 1	3M1	*CUL7*	2(5.3)	Primordial dwarfism and slender bones group
9	Chondrodysplasia punctata, X-linked dominant	CDPX2	*EBP*	1(2.6)	Chondrodysplasia punctata (CDP) group
10	Coffin-Siris syndrome 4	CSS4	*SMARCA4*	1(2.6)	Brachydactylies (with extraskeletal manifestations)
11	Larsen syndrome	LRS	*FLNB*	1(2.6)	Filamin group and related disorders
12	Pallister-Hall syndrome	PHS	*GLI3*	1(2.6)	Polydactyly-Syndactyly-Triphalangism group
13	Short-rib thoracic dysplasia 3 with or without polydactyly	SRTD3	*DYNC2H1*	1(2.6)	Ciliopathies with major skeletal involvement
14	Spondylometaphyseal dysplasia, Kozlowski type	SMDK	*TRPV4*	1(2.6)	TRPV4 group
15	Microphthalmia, syndromic 12	MCOPS12	*RARB*	1(2.6)	Non-skeletal dysplasias
16	Noonan syndrome 5	NS5	*RAF1*	1(2.6)	Non-skeletal dysplasias
17	Bosch-Boonstra-Schaaf optic atrophy syndrome	BBSOAS	*NR2F1*	1(2.6)	Non-skeletal dysplasias

^a^ Skeletal dysplasias classification based on the 2019 revision of the Nosology and Classification of Genetic Skeletal Disorders.

### 3.4 Variant analysis

Of the 43 fetuses diagnosed by ES, 31 with an autosomal dominant (AD) inheritance pattern, 10 with an autosomal recessive (AR) pattern and 2 with an X-linked pattern. Thirty-eight variants were identified in 20 different genes, including 26 missense variants, 6 non-sense variants, 2 frameshift variants, 3 in-frame ins/del variants, and 1 affects splice-site. Additionally, 31 (72.1%) variants were identified to be *de novo*, 7 (16.3%) variants were compound heterozygous inherited from the parents, three (7.0%) were homozygous mutations inherited from the parents, and two (4.7%) arose as a result of maternal mutation. Furthermore, fourteen (36.8%) novel SLBs-related variants were identified and analyzed in the present study (Table 4).

**TABLE 4:** Analysis of novel variants identified in this study.

Variant					Frequency		Pathogenicity scores	
Gene	Nucleotide	Protein	Variant tpyes	Classification	GnomAD exomes	GnomAD genomes	Revel	Conservation score (GERP)
*COL2A1*	NM_001844:c.1439G>A	NP_001835:p.G480E	missense	Likely Pathogenic	NF	NF	0.9890	4.8699
*COL2A1*	NM_001844:c.3580G>C	NP_001835:p.G1194R	missense	Likely Pathogenic	NF	NF	0.9480	5.1100
*CUL7*	NM_001168370:c.3333del	NP_001161842:p.F1112Sfs*61	frameshift	Likely Pathogenic	NF	NF	NA	4.9200
*CUL7*	NM_001168370:c.4855C>T	NP_001161842:p.Q1619*	non-sense	Likely Pathogenic	0.00000399	NF	NA	5.5000
*DYNC2H1*	NM_001080463:c.10526T>G	NP_001073932:p.M3509R	missense	Uncertain Significance	NF	NF	0.6039	6.0599
*DYNC2H1*	NM_001080463:c.6808C>T	NP_001073932:p.R2270*	non-sense	Likely Pathogenic	0.00000403	NF	NA	5.6999
*DYNC2H1*	NM_001080463:c.9977G>C	NP_001073932:p.R3326P	missense	Uncertain Significance	NF	NF	0.7170	5.6399
*GLI3*	NM_000168:c.3172C>T	NP_000159:p.R1058*	non-sense	Likely Pathogenic	NF	NF	NA	5.4699
*HSPG2*	NM_005529:c.2397_2399del	NP_005520:p.F800del	inframe	Uncertain Significance	NF	NF	NA	5.3299
*RARB*	NM_000965:c.1160G>A	NP_000956:p.R387H	missense	Likely Pathogenic	NF	NF	0.7089	5.8000
*SEC23B*	NM_001172745:c.1327_1338del	NP_001166216:p.G443_S446del	inframe	Uncertain Significance	NF	NF	NA	5.0700
*SEC23B*	NM_001172745:c.466A>G	NP_001166216:p.S156G	missense	Uncertain Significance	NF	NF	0.7250	4.9000
*XYLT1*	NM_022166:c.2456G>T	NP_071449:p.G819V	missense	Uncertain Significance	NF	NF	0.7049	4.9800
*ZC4H2*	NM_018684:c.635C>T	NP_061154:p.S212F	missense	Uncertain Significance	NF	NF	0.1689	5.4200

NF, not found; NA, not available.

### 3.5 Follow-up assessments

Clinical follow-up assessments were able to be performed in 91 cases (96.8%). Of 38 diagnosed cases, 37 fetuses underwent termination of pregnancy (TOP), and one infant (Case 35) with genetic abnormality died 2 months after birth. Among the fetuses of TOP, seven fetuses were autopsied and pathologically confirmed (Table 1). In addition, for the 5 cases with variants of uncertain significance, three cases with a specific diagnosis suspected or fetal FL significantly short (−3.9 SDs) underwent TOP, one case (Case 41) was healthy without clinical defects at the age of 3 years, and the one case refused follow up. Of the 51 fetuses with no prenatally detected genetic abnormalities, 5 were of incomplete follow-up data and 46 had outcome data ascertained. Twenty-three of 51 fetuses with multi-system abnormalities or with FL significantly short but without identified genetic abnormalities underwent TOP. Sixteen cases (16/46, 34.8%) were healthy and of no short stature at the age of follow-up. Seven (7/46, 15.2%) cases were followed up with clinical manifestations such as hypertonia, hypospadias, bilateral ventriculomegaly, bilateral talipes equinovarus, aortic stenosis, etc., (Table 5). One case was stillbirth with maternal gestational hypertension. Furthermore, among the fetuses without genetic abnormalities, the rate of TOP was significantly lower in FL 2-4 SDs below GA group compared with FL below −4 SDs group (36.8% [14/38] vs. 75.0% [9/12], *p* = 0.021). 6 of 20 children in FL 2–4SDs below GA group were associated with clinical abnormalities at the age of follow-up, while 1 out of 3 children was found to have clinical abnormalities in FL below -4SDs group (Table 5).

**TABLE 5:** Characteristics of clinically significant perinatal and postnatal conditions in 30 liveborns cases in whom follow up was ascertained.

Case^a^	Z-score	Other anomalies	Perinatal conditions				Postnatal conditions			
			Delivery modes	GA(w)	Sex	BW(kg)	Age of follow-up	Height(cm)	Weight(kg)	Clinical conditions and treatment
35	−2.867	Thickened nuchal skin fold, ventricular hypertrophy, battledore placenta	Unknown	Unknown	Unknown	Unknown	Dead at 2 months	-	-	-
41	−3.499	Tricuspid regurgitation	CS	35 + 6	Girl	2.1	3y	98	15	Normal
42	−2.963	Ventricular septal defect, narrow thorax	Unknown	Refuse follow-up	-	-	-	-	-	-
68	−5.068	Cardiomegaly, pericardial effusion	CS	35 + 3	Boy	1.58	2y2m	80	9.5	Normal
69	−2.078	No	CS	40 + 5	Girl	2.7	1y7m	82	10.5	Normal
70	−2.872	Abnormality of the thoracic spine	CS	39 + 1	Boy	2.9	1y11m	91	13	Mild delay in language and motor development
71	−2.248	Decreased head circumference	VD	29 + 4	Girl	1	1y6m	74	8.2	Hypertonia
72	−2.832	Polyhydramnios, echogenic fetal bowel	VD	Refuse follow-up	-	-	-	-	-	-
74	−2.148	No	CS	36 + 4	Boy	2	2y3m	84	9.5	Normal
75	−3.455	Persistent left superior vena cava	CS	35 + 4	Boy	1.5	2y8m	No response	No response	No response
76	−2.872	Increased fetal cardio thoracic ratio, large placenta	VD	39	Boy	2.25	2y5m	85	9.5	Hypospadias with surgical treatment, normal heart
77	−4.801	No	CS	37	Boy	1.5	1y10m	75	11	Normal
79	−2.166	Persistent left superior vena cava	VD	37 + 3	Boy	2.33	2y10m	80	10	Normal
80	−2.426	No	VD	38 + 6	Boy	3.5	4y1m	97	15	Normal
81	−2.364	No	VD	Refuse follow-up	-	-	-	-	-	-
82	−2.27	No	CS	39 + 3	Girl	2.55	3y5m	103	16.5	Normal
83	−2.174	No	CS	40 + 2	Girl	3.5	2y	83.4	11	Normal
84	−3.455	Battledore placenta, large placenta	CS	37 + 4	Girl	2.25	2y4m	85	10	Normal
86	−2.236	Polyhydramnios	CS	39 + 1	Girl	3.4	2y6m	88	11.5	Normal
87	−2.128	No	CS	38 + 1	Girl	2.65	1y9m	78	9	Normal
88	−2.356	No	CS	38 + 1	Girl	2.75	1y9m	No response	No response	No response
89	−2.319	No	CS	36 + 1	Girl	2.12	2y1m	84	11.3	Normal
45	−2.067	Increased nuchal translucency (4.6 mm)	VD	39 + 4	Boy	3.25	1y8m	84	10.2	Normal
47	−2.287	Hyperechogenic kidneys	CS	38 + 2	Boy	2.7	1y6m	77	8	Normal
48	−2.93	Increased nuchal translucency (3.4 mm)	VD	38 + 2	Girl	2.81	1y11m	No response	No response	No response
55	−6.061	Hypospadias	CS	33 + 6	Boy	1.12	2y	90	11	hypospadias with surgical treatment
56	−2.569	Dilation of lateral ventricles, hyperechogenic kidneys	CS	38 + 6	Boy	3.96	1y4m	77	9.8	Bilateral ventriculomegaly, normal kidneys, currently hospitalized for pneumonia
57	−3.172	Decreased head circumference, bilateral talipes equinovarus, separation of renal pelvic	CS	39 + 3	Girl	2.7	1y6m	76.8	9.5	Bilateral talipes equinovarus and treated
65	−3.172	Aortic valve stenosis, single umbilical artery, hypoplasia of the nasal bone	VD	39 + 4	Girl	2.65	1y8m	80+	10	Aortic stenosis without treatment
67	−2.872	Micrognathia	CS	35	Girl	2.8	4y9m	102	14	Normal

GA, gestational age; BW, birth weight; CS, cesarean section; VD, vaginal delivery.

^a^ Case 35 was identified with a pathogenic variant; Case 41 and Case 42 harbored variants of uncertain significance; all other cases were of negative results of genetic tests.

## 4 Discussion

Fetal short long bones bring diagnostic challenges for clinicians. Although in most cases, it may be constitutional, it is also recognized as a marker of chromosomal abnormalities or skeletal dysplasias. The vast majority of skeletal dysplasias are associated with monogenic abnormalities (Snijders et al., 2000; Vermeer and Bekker, 2013; Li et al., 2021). We performed a cohort study to identify the genetic causes for fetal SLBs and retrospectively evaluate the additional diagnostic yield of ES after a negative result of CMA or karyotype analysis. Besides of genetic diagnosis, we also performed long-term follow-ups to assess the prognoses and outcomes in fetuses with SLBs.

Prenatal ES identified well-described genetic causes in 38 out of 94 cases (40.4%), and VUS in 5 cases, rendering a total diagnostic yield of 45.74%. Compared with our findings, three recent cohort studies reported a significantly higher diagnostic rate by prenatal ES, which was 80% (12 out of 15), 85% (11 out of 13), and 70% (21 out of 30), respectively (Liu et al., 2019; Peng et al., 2021; Tang et al., 2021). Many aspects could affect the detection rate of ES, such as the selected criteria of the study, the number of cases, proband-only or trio ES, and so on (Tang et al., 2021). The number of cases in previous studies were comparatively small (≤30 cases), and small study number may cause relatively high selected bias. In some previous studies, fetal growth restriction and some non-genetic causes were excluded, and may also bring in selected bias. It was reported that trio-ES had a higher diagnostic rate; fetuses with multiple anomalies also had a higher diagnostic rate, and when testing single structural anomalies, a particular organ system may have a higher diagnostic yield (Petrovski et al., 2019). In our study, ES yielded 27.3% (6 out of 22) in the isolated SLBs group and 44.4% (32 out of 72) in the non-isolated SLBs group. For fetuses with SLBs and other skeletal abnormalities (femur fracture, femoral bowing, spinal dysplasia, bilateral talipes equinovarus, et al.), the diagnostic rate was 59.4% (19 out of 32), and for fetuses with SLBs and non-skeletal abnormalities (increased nuchal translucency, tricuspid regurgitation, cerebellar dysplasia, polyhydramnios, et al.) the diagnostic rate was 32.5% (13 out of 40). The finding is consistent with previous studies, suggesting that fetuses with SLBs and other skeletal abnormalities were the most common associations with skeletal dysplasias (Papageorghiou et al., 2008). Therefore, when prenatal ultrasound scans show that the fetal femur is 2 or more SDs below the normal range, the length of the other long bones should be measured and a thorough skeletal assessment should be performed to determine whether there are other skeletal morphological abnormalities (Liu et al., 2019).

Moreover, diverse degree of femoral shortening may confer different associated genetic risks (Li et al., 2021). Of the 94 fetuses that underwent ES, 54 fetuses had Z-scores between −2 SDs and −4 SDs, and 40 fetuses had Z-scores below −4 SDs. The diagnostic rate was 16.7% (9 out of 54) in fetuses with Z-scores between −2 SDs and −4 SDs, and 72.50% (29 out of 40) in fetuses with Z-scores below −4 SDs. In the present study, the degree of femoral shortening is correlated with the rate of adverse pregnancy outcomes. In fetuses with FL below −4 SDs, the abnormal pregnancy outcomes were significantly higher. Therefore, prenatal ES can be used as an effective clinical diagnostic platform for fetuses with short long bones, especially in those with multiple congenital anomalies or severely short limbs.

In the present study, 38 variants affecting 20 genes including *FGFR3*, *CUL7*, *COL1A2*, *COL2A1*, *COL1A1*, *DYNC2H1*, *PPIB*, *PYCR1*, *EBP*, *HSPG2*, *FLNB*, *GLI3*, *NR2F1*, *RAF1*, *RARB*, *SEC23B*, *SMARCA4*, *TRPV4*, *XYLT1*, and *ZC4H2* were found to be associated with monogenic disorders. Of 38 diagnosed cases, 92.1% (35/38) were diagnosed as genetic skeletal dysplasias, including 14 different diseases that are classified into 10 groups according to the 2019 revision of the Nosology and Classification of Genetic Skeletal Disorders (Mortier et al., 2019). Among the fetuses with genetic skeletal dysplasias, 42.1% (16/38) contained variants in *FGFR3*, which were categorized into *FGFR3* chondrodysplasia group based on the Nosology and Classification of Genetic Skeletal Disorders. Moreover, 13.2% (5/38) of fetuses contained variants in *COL1A1*/*COL1A2*, 5.3% (2/38) contained variants in *PPIB*, and one fetus contained variants in *PYCR1*. These genes were categorized into the group of osteogenesis imperfecta and decreased bone density. In addition, 7.9% (3/38) of the fetuses contained variants in *COL2A1* that were diagnosed as achondrogenesis, type II. 5.3% (2/38) of the fetuses harbored variants in *CUL7* diagnosed with 3-M syndrome 1, which would lead to primordial dwarfism. These findings are in line with the high genetic variability of fetal SLBs, and also highlights that pathogenic variants in *FGFR3* and collagen genes are the most common genetic causes for SLBs, as well as for skeletal dysplasias.

Additionally, fourteen variants identified in this study were novel. Novel pathogenic variants identified may broaden the mutation spectrum for the disorders and contributes to clinical consultation and subsequent pregnancy examination.

Fibroblast growth factor receptor 3 (*FGFR3*) is one of four distinct membrane-spanning tyrosine kinases that serve as high-affinity receptors for a number of fibroblast growth factors and play essential roles in skeletal development (Ornitz et al., 2015; Peng et al., 2021). Variants in *FGFR3* have been associated with at least 10 different skeletal disorders (Mortier et al., 2019; Peng et al., 2021). In the present study, 11 cases (Case 13–14, Case 16–18, Case 20–22, Case 24–25, Case 28) had a hotspot mutation, c.1138G>A in *FGFR3*, that would lead to achondroplasia (MIM_100,800) (Hafner et al., 2006; Natacci et al., 2008). Two cases (Case 23, Case 27) carried an identical variant c.742C>T in *FGFR3* gene, which has been detected in different populations (Chen et al., 2001; Tonni et al., 2010; Tang et al., 2021). The variant c.746C>G and c.1118A>G in *FGFR3* were identified in one case each (Case 15, Case 26). Variants c.742C>T, c.746C>G, and c.1118A>G have been associated with thanatophoric dysplasia, type I (MIM_187,600) (Brodie et al., 1999). Case 19 harbored a variant c.1620C>A (p.N540K) in the TK-1 domain of *FGFR3*, which is a common cause of hypochondroplasia (MIM_146,000) (Mortier et al., 2019).

Mutations in the type I collagen coding genes (*COL1A1* and *COL1A2*) affecting collagen quantity or structure count for approximately 85% of osteogenesis imperfecta (OI) cases (Marini et al., 2017; Tournis and Dede, 2018; Peng et al., 2021). The helical domains contain Gly-X-Y triplets where glycine substitutions are the most frequent cause of OI (Marini et al., 2017; Peng et al., 2021). In five cases of the present study, variant c.3118G>A, *COL1A2* c.3277G>A, c.3034G>A and c.1801G>A in *COL1A1* were identified, causing a glycine in the helical domain of type 1 collagen was substituted. These variants may disrupt type 1 collagen functions, and therefore lead to skeletal dysplasias. Besides, Case 1 harbored a variant c.957 + 1G>T in *COL1A1*, that was predicted to disrupt splicing by SpliceAI. *COL2A1* gene encodes type II collagen that plays a role in the regulation of intramembranous and endochondral ossification. Heterozygous *COL2A1* variants are associated with a spectrum of dwarfism and skeletal malformation diseases (Zhang R et al., 2020). In the present study, 2 novel likely pathogenic mutations (c.1439G>A and c.3580 G>C) and 1 known likely pathogenic mutation (c. 3472G>A) were identified in *COL2A1* (Table 1). These findings further indicate the high genetic variability of fetal SLBs and also highlight the importance of molecular diagnosis for fetuses with SLBs.

In the present study, we performed a long-term follow-up for up to 5 years after birth. The pregnancy outcomes for fetuses with FL 2- 4 SDs below GA were significantly better than those with FL below −4 SDs. Consistent with previous studies, the severe femoral shortening was the main reason for TOP in our cohort with fetal SLBs (Li et al., 2021). Fifty-six cases in the present study did not have a definitive molecular diagnosis, and 12 of them (21.4%) with Z-scores below −4SDs. The negative ES results could be attributed to limited phenotypes or the limitations of ES (Tang et al., 2021). For prenatal ES, several aspects could affect the detection rate, especially incomplete prenatal phenotypes (Aarabi et al., 2018; Tang et al., 2021). Meanwhile, many aspects should be considered, such as ethical concerns, analysis of variants of unknown significance, and secondary findings (Best et al., 2018; Monaghan et al., 2020; Tang et al., 2021). In the future, with the development of fetal-specific phenotype-genotype database, and increased studies on the molecular mechanisms of genes associated with SLBs, the uncertainty in cases will become less frequent (Aarabi et al., 2018; Tang et al., 2021). Furthermore, the causative variants may reside in the non-coding-regulatory or deep-intronic regions, and may not be detected by ES, but could be identified by whole genome sequencing in the future (Tang et al., 2021). Additionally, our study has some limitations. For instance, this is a single-center study with a relatively small case number and selected population. Prospective multicenter studies with large sample sizes are needed to obtain more reliable data (Li et al., 2021). In some cases, telephone follow-up may induce recall bias (Peng et al., 2021).

## 5 Conclusion

Prenatal exome sequencing analysis facilitates genetic diagnosis and improves the management of pregnancies with fetal short long bones detected by ultrasonography especially in those with multiple congenital anomalies or severely short limbs. Additionally, novel pathogenic variants identified may broaden the mutation spectrum for the disorders and contributes to clinical consultation and subsequent pregnancy examination.

## Data Availability

The datasets presented in this study have been uploaded in online repositories. The names of the repository/repositories and accession number(s) can be found below: Genome Sequence Archive (Genomics, Proteomics, and Bioinformatics 2021) in National Genomics Data Center (Nucleic Acids Res 2021), China National Center for Bioinformation/Beijing Institute of Genomics, Chinese Academy of Sciences (GSA-Human: HRA003147).

## Abbreviations

SLBs, short long bones; SDs, standard deviations; CMA, chromosomal microarray analysis; NGS, next-generation sequencing; FL, femur length; MAF, minor allele frequency; CNV, Copy number variations; AD, autosomal dominant; AR, autosomal recessive; VUS, Variants of uncertain clinical significance.
